# Risk Models to Predict Chronic Kidney Disease and Its Progression: A Systematic Review

**DOI:** 10.1371/journal.pmed.1001344

**Published:** 2012-11-20

**Authors:** Justin B. Echouffo-Tcheugui, Andre P. Kengne

**Affiliations:** 1 Hubert Department of Global Health, Rollins School of Public Health, Emory University, Atlanta, Georgia, United States of America; 2 NCRP for Cardiovascular and Metabolic Diseases, South African Medical Research Council, Cape Town, South Africa; 3 Department of Medicine, Faculty of Health Sciences, University of Cape Town, Cape Town, South Africa; 4 The George Institute for Global Health, Sydney, Australia; 5 Julius Center for Health Sciences and Primary Care, University Medical Center Utrecht, Utrecht, The Netherlands; Instituto Mario Negri, Italy

## Abstract

**Background:**

Chronic kidney disease (CKD) is common, and associated with increased risk of cardiovascular disease and end-stage renal disease, which are potentially preventable through early identification and treatment of individuals at risk. Although risk factors for occurrence and progression of CKD have been identified, their utility for CKD risk stratification through prediction models remains unclear. We critically assessed risk models to predict CKD and its progression, and evaluated their suitability for clinical use.

**Methods and Findings:**

We systematically searched MEDLINE and Embase (1 January 1980 to 20 June 2012). Dual review was conducted to identify studies that reported on the development, validation, or impact assessment of a model constructed to predict the occurrence/presence of CKD or progression to advanced stages. Data were extracted on study characteristics, risk predictors, discrimination, calibration, and reclassification performance of models, as well as validation and impact analyses. We included 26 publications reporting on 30 CKD occurrence prediction risk scores and 17 CKD progression prediction risk scores. The vast majority of CKD risk models had acceptable-to-good discriminatory performance (area under the receiver operating characteristic curve>0.70) in the derivation sample. Calibration was less commonly assessed, but overall was found to be acceptable. Only eight CKD occurrence and five CKD progression risk models have been externally validated, displaying modest-to-acceptable discrimination. Whether novel biomarkers of CKD (circulatory or genetic) can improve prediction largely remains unclear, and impact studies of CKD prediction models have not yet been conducted. Limitations of risk models include the lack of ethnic diversity in derivation samples, and the scarcity of validation studies. The review is limited by the lack of an agreed-on system for rating prediction models, and the difficulty of assessing publication bias.

**Conclusions:**

The development and clinical application of renal risk scores is in its infancy; however, the discriminatory performance of existing tools is acceptable. The effect of using these models in practice is still to be explored.

*Please see later in the article for the Editors' Summary*

## Abstract 2

A systematic review of risk prediction models conducted by Justin Echouffo-Tcheugui and Andre Kengne examines the evidence base for prediction of chronic kidney disease risk and its progression, and suitability of such models for clinical use.

## Editors' Summary

**Background:**

Chronic kidney disease (CKD)—the gradual loss of kidney function—is increasingly common worldwide. In the US, for example, about 26 million adults have CKD, and millions more are at risk of developing the condition. Throughout life, small structures called nephrons inside the kidneys filter waste products and excess water from the blood to make urine. If the nephrons stop working because of injury or disease, the rate of blood filtration decreases, and dangerous amounts of waste products such as creatinine build up in the blood. Symptoms of CKD, which rarely occur until the disease is very advanced, include tiredness, swollen feet and ankles, puffiness around the eyes, and frequent urination, especially at night. There is no cure for CKD, but progression of the disease can be slowed by controlling high blood pressure and diabetes, both of which cause CKD, and by adopting a healthy lifestyle. The same interventions also reduce the chances of CKD developing in the first place.

**Why Was This Study Done?:**

CKD is associated with an increased risk of end-stage renal disease, which is treated with dialysis or by kidney transplantation (renal replacement therapies), and of cardiovascular disease. These life-threatening complications are potentially preventable through early identification and treatment of CKD, but most people present with advanced disease. Early identification would be particularly useful in developing countries, where renal replacement therapies are not readily available and resources for treating cardiovascular problems are limited. One way to identify people at risk of a disease is to use a “risk model.” Risk models are constructed by testing the ability of different combinations of risk factors that are associated with a specific disease to identify those individuals in a “derivation sample” who have the disease. The model is then validated on an independent group of people. In this systematic review (a study that uses predefined criteria to identify all the research on a given topic), the researchers critically assess the ability of existing CKD risk models to predict the occurrence of CKD and its progression, and evaluate their suitability for clinical use.

**What Did the Researchers Do and Find?:**

The researchers identified 26 publications reporting on 30 risk models for CKD occurrence and 17 risk models for CKD progression that met their predefined criteria. The risk factors most commonly included in these models were age, sex, body mass index, diabetes status, systolic blood pressure, serum creatinine, protein in the urine, and serum albumin or total protein. Nearly all the models had acceptable-to-good discriminatory performance (a measure of how well a model separates people who have a disease from people who do not have the disease) in the derivation sample. Not all the models had been calibrated (assessed for whether the average predicted risk within a group matched the proportion that actually developed the disease), but in those that had been assessed calibration was good. Only eight CKD occurrence and five CKD progression risk models had been externally validated; discrimination in the validation samples was modest-to-acceptable. Finally, very few studies had assessed whether adding extra variables to CKD risk models (for example, genetic markers) improved prediction, and none had assessed the impact of adopting CKD risk models on the clinical care and outcomes of patients.

**What Do These Findings Mean?:**

These findings suggest that the development and clinical application of CKD risk models is still in its infancy. Specifically, these findings indicate that the existing models need to be better calibrated and need to be externally validated in different populations (most of the models were tested only in predominantly white populations) before they are incorporated into guidelines. The impact of their use on clinical outcomes also needs to be assessed before their widespread use is recommended. Such research is worthwhile, however, because of the potential public health and clinical applications of well-designed risk models for CKD. Such models could be used to identify segments of the population that would benefit most from screening for CKD, for example. Moreover, risk communication to patients could motivate them to adopt a healthy lifestyle and to adhere to prescribed medications, and the use of models for predicting CKD progression could help clinicians tailor disease-modifying therapies to individual patient needs.

**Additional Information:**

Please access these websites via the online version of this summary at http://dx.doi.org/10.1371/journal.pmed.1001344.

- This study is further discussed in a *PLOS Medicine* Perspective by Maarten Taal
- The US National Kidney and Urologic Diseases Information Clearinghouse provides information about all aspects of kidney disease; the US National Kidney Disease Education Program provides resources to help improve the understanding, detection, and management of kidney disease (in English and Spanish)
- The UK National Health Service Choices website provides information for patients on chronic kidney disease, including some personal stories
- The US National Kidney Foundation, a not-for-profit organization, provides information about chronic kidney disease (in English and Spanish)
- The not-for-profit UK National Kidney Federation support and information for patients with kidney disease and for their carers, including a selection of patient experiences of kidney disease
- World Kidney Day, a joint initiative between the International Society of Nephrology and the International Federation of Kidney Foundations, aims to raise awareness about kidneys and kidney disease

## Introduction

Chronic kidney disease (CKD) is increasingly common in the US and worldwide [1],[2]. Related complications, including end-stage renal disease (ESRD) and cardiovascular disease (CVD), have major public health and economic implications [1]–[3]. Screening for CKD has been somewhat controversial in the absence of direct evidence from a randomized clinical trial [4]. However, early identification of individuals with CKD, especially targeting populations with a high risk for CKD and related adverse outcomes [5], followed by the implementation of evidence-based interventions can slow or prevent the progression to advanced stages of the disease, reduce the risk of CVD and other complications of decreased glomerular filtration rate (GFR), and improve survival and quality of life [6]. However, large proportions of individuals with CKD remain undiagnosed and, as a consequence, are not benefiting from those interventions. For instance, in the US, awareness of CKD in the general population remains very low [1]. During the 1999–2004 period, the proportion of US adults with stage 3 CKD who reported being aware of their status was only 11.6% in men and 5.5% in women. Even among men with stage 3 CKD and elevated albuminuria, awareness of weak or failing kidneys was only 22.8%. Among those with stage 4 CKD, the corresponding percentage was 42% for both men and women [1]. In clinical settings, awareness levels are also low. Data from the US National Kidney Foundation's Kidney Early Evaluation Program, for the 2000–2009 period, indicate that only 9% of patients with CKD are aware of their diagnosis [7].

Strategies for early identification and treatment of people with CKD are therefore needed worldwide. The use of complex and potentially expensive detection strategies may prevent those at risk from deriving the benefits of preventative interventions, especially in settings where renal replacement therapy is not readily available. Several risk factors that are independently associated with the occurrence of CKD and easily assessable in routine clinical settings have been incorporated in model equations for predicting the occurrence of CKD or progression in people already diagnosed with CKD. These models have utility even in the context of automatic reporting of the estimated GFR (eGFR). Indeed, recent data indicate that referral to a nephrologist by primary care physicians as the result of making eGFR available mostly occurs for certain subgroups in the population (women and elderly), and a high proportion of referrals are inappropriate [8].

The use of risk models is very attractive and likely cost-effective for large-scale CKD risk stratification, and would allow the identification of all the segments of the population that would benefit the most from CKD detection. To this end, it is very important that existing models are not methodologically flawed, and that they provide accurate estimates of the CKD risk in different populations.

To date, there has been no effort, to our knowledge, to provide decision makers and healthcare providers with a balanced account of the performance of existing CKD risk models. We therefore systematically reviewed studies of risk equations to predict CKD or its progression, with the objectives of summarizing evidence on their performance and exploring methodological issues surrounding their development and validation and application.

## Methods

We performed literature searches to identify all risk models developed to predict the presence/occurrence of CKD, or to predict the progression of CKD in those with the disease. We also searched for all studies that applied existing CKD risk models either in the population from which the model was developed or in different populations, and, lastly, we searched for all impact studies and clinical practice guidelines that incorporated existing CKD risk models.

### Model Development and Validation Studies

#### Data sources and search strategy

We searched the PubMed MEDLINE and Embase databases from 1 January 1980 to 20 June 2012, for English- or French- language studies of CKD risk prediction model development and/or validation. We used a combination of search terms related to CKD and prediction. The search strategies are provided in detail in Texts S2 and S3. In addition, we manually searched the reference lists of eligible studies and relevant reviews, and traced studies that had cited them through the ISI Web of Science to find additional published and unpublished data.

#### Study selection

**Figure 1:**
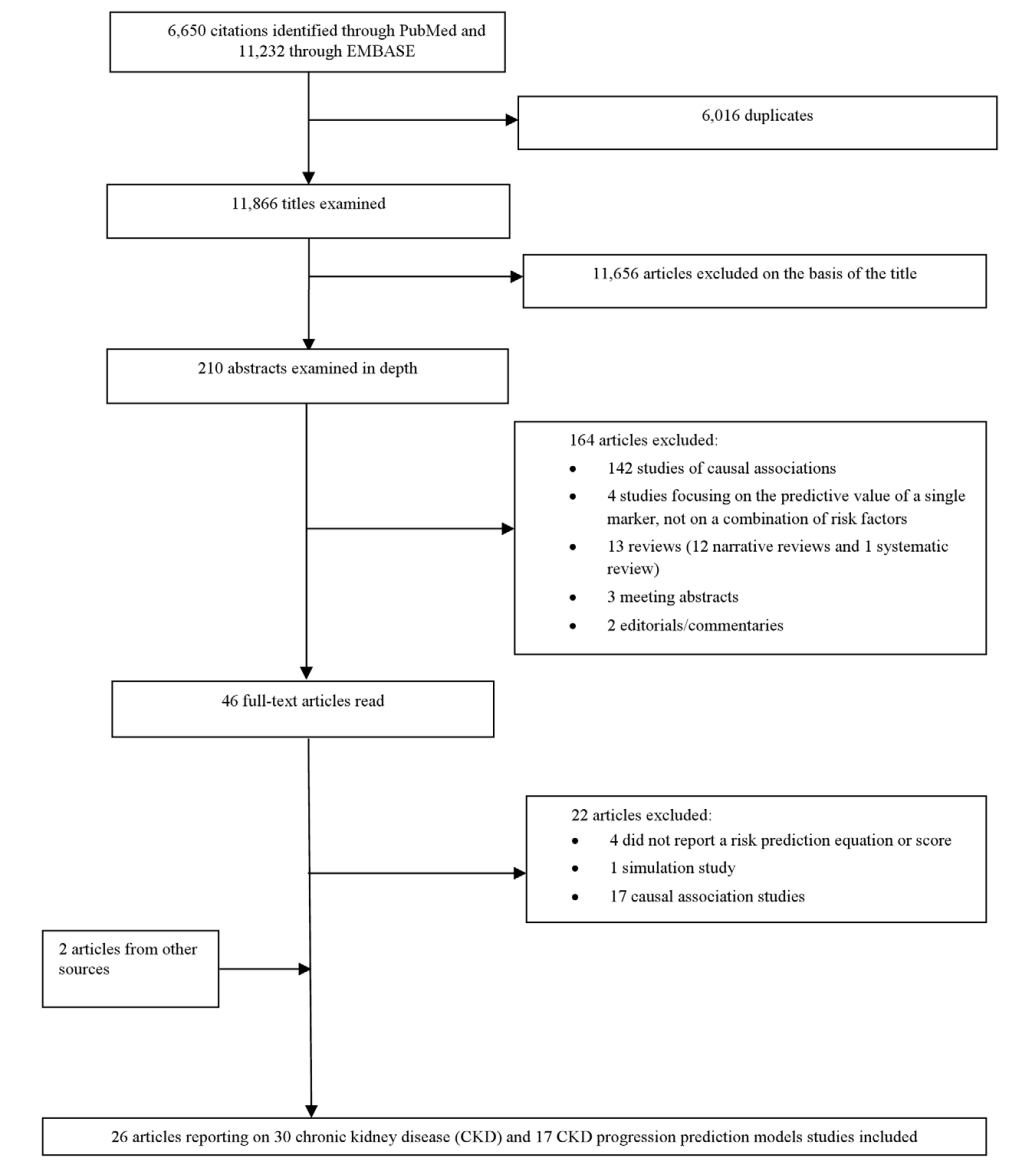
Article selection process.

Two evaluators (J. B. E. and A. P. K.) independently identified articles and sequentially screened them for inclusion (Figure 1). Where necessary, the full text of articles and/or supplemental materials (tables and appendices) was reviewed before deciding on inclusion. Disagreements were solved by consensus between both authors.

Eligible articles had to report a risk assessment tool (equation and/or score) for predicting CKD or its progression, derived in adult human populations. Reporting of quantitative measures of the performance of tools was preferable, but not necessary for inclusion. The reported metrics of evaluation of predictive ability could be the area under the receiver operating characteristic curve (AUC) or C-statistic, reclassification percentage, net reclassification improvement (NRI), or integrated discrimination improvement index (IDI). These metrics are recognized and used for the assessment of prediction models [9],[10]. We excluded studies that reported only measures of association between risk factors and CKD without information on the beta coefficients of variables included in a prediction equation, and simulation studies.

#### Data extraction and quality assessment

Two reviewers (J. B. E. and A. P. K.) independently conducted the data extraction and quality assessment. We did not use a particular framework for quality assessment, as there is no consensus on a quality assessment framework for risk prediction models. Consequently, we did not develop a formal protocol for the review (Text S1). From each study, we extracted data on study design, setting, population characteristics, the number of patients in the derivation and validation cohorts, the number of participants with the outcome of interest, the number of candidate variables tested as predictors, and the number and list of those variables included in the final model, as well as the type of statistical model used. For the discriminative performance of models, we extracted information on the AUC or C-statistic, which indicates the ability of a risk model to rank-order individuals' risks. To describe model calibration, we extracted data on the difference between the observed and predicted rates of CKD, as well as the *p*-value of the corresponding test statistic. Measures of calibration assess the ability of a risk prediction model to predict accurately the absolute level of risk that is subsequently observed.

For the assessment of reclassification, we extracted the NRI and IDI values, and the accompanying 95% CIs and *p*-values, when available. Reclassification analyses generally indicate the proportion of individuals who are reclassified from one risk stratum (based on estimated risk provided from a first model) to a different risk stratum (based on estimated risk from a different model, or a model that has additional variables compared with the first model). The IDI measures the extent to which the use of a new risk marker correctly revises upward the predicted risk of individuals who experienced the event of interest and correctly revises downward the predicted risk of individuals who did not experience the event.

#### Data synthesis

Given the wide range of metrics used for the assessment of the predictive ability of CKD risk models, and the heterogeneity in both the risk factors used for prediction and their number, as well as the study designs, we opted to conduct a narrative synthesis of the evidence instead of a meta-analysis.

### Impact Studies and Implementation of Risk Models in Guidelines

Impact studies were captured by (1) scanning those publications identified through the search strategy for model development and validation, and (2) applying the search strategy for impact studies proposed by Reilly and Evans [11], which combines the model's acronym, name of the cohort, or first author with a specific search term (Text S3). We searched relevant clinical practice guidelines to investigate the implementation of CKD prediction models in countries in which such models have been developed. In the absence of validated strategies for these types of searches, we targeted guidelines (when available in English language) compiled by a selection of organizations known to be involved in issues relating to kidney diseases, including the American Society of Nephrology (http://www.asn-online.org), the US National Kidney Foundation [12], the UK National Institute for Health and Clinical Excellence [13], the International Society of Nephrology [14], the European Renal Association–European Dialysis and Transplant [15], the Canadian Society of Nephrology [16], Kidney Disease: Improving Global Outcomes [17], The Korean Society of Nephrology (http://www.ksn.or.kr/english/), the Japanese Society for Dialysis Therapy [18], The Japan Association of Chronic Kidney Disease Initiatives (J-CKDI) [19], and the Taiwan Society of Nephrology [20].

## Results

Figure 1 describes the study selection process. Of the citations identified through searches, 210 abstracts were selected for in-depth evaluation, and 46 full-text publications were reviewed. After all exclusions, 26 articles, reporting on 30 CKD prediction risk scores and 17 CKD progression risk scores, met the eligibility criteria and were included in the review.

### CKD Prediction Risk Scores

Table 1 summarizes data from studies that developed CKD risk prediction models. Five of the 30 CKD risk prediction models were developed using cross-sectional data (thus, prevalent CKD) [21]–[24], and the remaining models were based on cohort studies.

**Table 1:** Development of risk models for predicting chronic kidney disease.

Study	Country/Ethnicity	Design/Setting	Candidate Variables (*n*)	Risk factors included	*n* Total/*n* Outcomes	Age (Years)	Outcomes Predicted	Time Horizon (Years)^a^	Discrimination AUC	Calibration	Method of Internal Validation	Type of Model
Bang et al. 2007 [21]—SCORED score	US/mixed	Cross-sectional/population-based	24	Age, sex, anemia, HTN, diabetes, Hx of CVD, Hx of CHF, PVD	8,530/601	20–85	CKD (GFR<60 ml/min/1.73 m^2^)—MDRD equation	NA	0.88	NR	Apparent	Logistic
									0.88	NR	Split-sample	
Kshirsagar et al. 2008 [35]—ARIC/CHS score 1	US/white and black	Prospective cohort/population-based	19	Age, anemia, sex, HTN, diabetes, PVD, Hx of CHF or CVD	9,470/1,605	45–64	CKD (GFR<60 ml/min/1.73 m^2^)—MDRD equation	4–9	0.69	HL test (*p*>0.2)	Apparent	Logistic
									0.68	NR	Split-sample	
Kshirsagar et al. 2008 [35]—ARIC/CHS score 2	US/white and black	Prospective cohort/population-based	19	Age, anemia, sex, HTN, diabetes, low HDL, PVD, Hx of CHF or CVD	9,470/1,605	45–64	CKD (GFR<60 ml/min/1.73 m^2^)—MDRD equation	4–9	0.70	HL test (*p*>0.2)	Apparent	Logistic
									0.70	NR	Split-sample	
Fox et al. 2010 [30]—Framingham score 1	US/white	Prospective cohort/population-based	NR	Age, sex	2,345/213	Mean: 56.6	CKD (GFR<60 ml/min/1.73 m^2^)—MDRD equation	9.5	0.776	HL χ^2^ = 8.20 (*p* = 0.41)	Apparent	Logistic
Fox et al. 2010 [30]—Framingham score 2	US/mainly white	Prospective cohort/population-based	NR	Age, sex, SBP, HTN, HTN treatment, smoking, BMI, HDL, diabetes, eGFR,	2,345/213	Mean: 56.6	CKD (GFR<60 ml/min/1.73 m2)—MDRD equation	9.5	0.81,	HL χ^2^ = 2.98 (*p* = 0.94)	Apparent	Logistic
Fox et al. 2010 [30]—Framingham score 3	US/mainly white	Prospective cohort/population-based	NR	Age, sex, SBP, HTN, HTN treatment, smoking, BMI, HDL, diabetes, eGFR, aldosterone, homocysteine	2,345/213	Mean: 56.6	CKD (GFR<60 ml/min/1.73 m^2^)—MDRD equation	9.5	0.82	HL χ^2^ = 3.48 (*p* = 0.90)	Apparent	Logistic
Hippisley-Cox and Coupland 2010 [34]—QKidney score	UK/mixed: white, black, South-Asian, Chinese	Prospective cohort//population-based	18	Age, ethnicity, deprivation, smoking, BMI, SBP, diabetes, rheumatoid arthritis, CVD, treated HTN, CHF, PVD, NSAID use, family Hx of KD, SLE, kidney stones	1,591,884 (775,091 women and 799,658 men)/23,786 (CKD); 1,266 (ESRD)	35–74	Moderate-severe CKD (kidney transplant, dialysis, nephropathy, persistent proteinuria, or eGFR<45 ml/min/1.73 m^2^) and ESRD (kidney transplant, dialysis, or eGFR<15 ml/min/1.73 m^2^)—MDRD equation	5	CKD stage: 0.88, men; 0.88, women/ESRD stage: 0.85, men; 0.84, women	NR	Apparent	Cox
Chien et al. 2010 [25]—Taiwan score 1	Taiwan/Chinese	Prospective cohort/population-based	12	Age, BMI, DBP, Hx of T2DM, stroke	5,168/190	Mean: 51.2	CKD (GFR reduced but ≥ 60 ml/min/1.73 m^2^)—MDRD equation	4	0.77	HL test (*p*>0.10).	Apparent	Cox
Chien et al. 2010 [25]—Taiwan score 2	Taiwan/Chinese	Prospective cohort/population-based	12	Age, BMI, DBP, Hx of T2DM, stroke, uric acid, postprandial glucose, HbA1c, proteinuria	5,168/190	Mean: 51.2	CKD (GFR reduced but ≥ 60 ml/min/1.73 m2)—MDRD equation	4	0.77	HL test (*p*>0.10)	Apparent	Cox
Halbesma et al. 2011 [48]—PREVEND score	Netherlands/white	Prospective cohort/population-based	18	Age, urinary albumin excretion, SBP, CRP, known HTN, eGFR	6,809/272	28–75	CKD (the most renal function decline [top 20% of the total population] and eGFR<60 ml/min/1.73 m^2^ at follow-up)—MDRD equation	6.4	0.84	NR	Apparent	Logistic
									0.84	NR	Bootstrap	
Ando et al. 2011 [26]—Japan/HIV score	Japan/Asian	Prospective cohort/clinic-based	8	Age, CD4 count, diabetes, proteinuria, eGFR	534 (HIV patients)/18	20–81	CKD (eGFR<60 ml/min/1.73 m^2^)—MDRD equation	1	0.84	NR	Apparent	Logistic
Blech et al. 2011 [22]—Israel score 1	Israel/white	Cross-sectional/clinic-based	NR	Age, sex, ethnicity, diabetes type and duration	1,274/556	Mean: 62.6	Diabetic nephropathy (microalbuminuria [0.03–0.3 g/g creatinine], proteinuria [>0.3 g/g creatinine]), or dialysis in the absence of any other unrelated renal disease	NA	0.58	NR	Apparent	Logistic
Blech et al. 2011 [22]—Israel score 2	Israel/white	Cross-sectional/clinic-based	NR	Five SNPs in five genes (HSPG2, NOS3, ADIPOR2, AGER, CCL5), age, sex, ethnicity, diabetes type and duration	1,274/556	Mean: 62.6	Diabetic nephropathy (microalbuminuria [0.03–0.3 g/g creatinine], proteinuria [>0.3 g/g creatinine]), or dialysis in the absence of any other unrelated renal disease	NA	0.67	NR	Apparent	Logistic
									0.63	NR	Split-sample	
Thakkinstian et al. 2011 [23]—Thailand score	Thailand/Asian	Cross-sectional/population-based	16	Age, diabetes, HTN, Hx of kidney stones	3,459/606	≥18	CKD (eGFR<60 ml/min/1.73 m^2^)—MDRD equation	NA	0.77	Bias observed versus predicted values: 0.045	Apparent	Logistic
									0.74.	NR	Bootstrap	
O'Seaghdha et al. 2012 [31]—Framingham score 3a	US/white	Prospective cohort/population-based	NR	Age, sex, cohort status, baseline eGFR, HTN, diabetes, proteinuria, 16 SNPs	2,489/270	28–62	CKD (eGFR<60 ml/min/1.73 m^2^)—MDRD equation		0.780, without genotype score; 0.781, with genotype score (difference: *p* = 0.2)	NR	Apparent	Logistic
O'Seaghdha et al. 2012 [31]—Framingham score 3b	US/mainly white	Prospective cohort/population-based	NR	Age, sex, 16 SNPs	2,489/270	28–62	CKD (eGFR<60 ml/min/1.73 m^2^)—MDRD equation	10	0.748, without genotype score; 0.751, with genotype score (difference: *p* = 0.3)	NR	Apparent	Logistic
O'Seaghdha et al. 2012 [32]—Framingham score 4a	US/mainly white	Prospective cohort/population-based	NR	Age, diabetes, HTN	2,490/229	45–64	CKD (eGFR<60 ml/min/1.73 m^2^)—MDRD equation	10	0.79.	NR	Apparent	Logistic
O'Seaghdha et al. 2012 [32]—Framingham score 4b	US/mainly white	Prospective cohort/population-based	NR	Age, diabetes, HTN, eGFR category	2,490/229	45–64	CKD (eGFR<60 ml/min/1.73 m^2^)—MDRD equation	10	0.81	NR	Apparent	Logistic
O'Seaghdha et al. 2012 [32]—Framingham score 4c	US/mainly white	Prospective cohort/population-based	NR	Age, HTN, diabetes, baseline eGFR, albuminuria	2,490/229	45–64	CKD (eGFR<60 ml/min/1.73 m^2^)—MDRD equation	10	0.81	HL χ^2^ = 7.27; (*p* = 0.60)	Apparent	Logistic
									0.79	NR	Bootstrap	
Alssema et al. 2012 [49]—Rotterdam-Hoorn score	Netherlands/white	Prospective cohort/population-based	9	Age, BMI, waist circumference, HTN treatment, current smoking, parent and/or sibling with CVD (age<65 y), parent and/or sibling with diabetes	6,019/366	28–85	CKD (eGFR<60 ml/min/1.73 m^2^)—MDRD equation	7	0.82, men; 0.81, women	HL χ^2^ = 7.6 (*p* = 0.48), men; 6.3 (*p* = 0.62), women	Apparent	Logistic
									0.80, men; 0.82, women		Bootstrap	
Kwon et al. 2012 [24]—Korean risk score	Korea/Asian	Cross-sectional/population-based	16	Age, sex, anemia, HTN, diabetes, CVD, proteinuria	6,565/100	≥19	CKD (eGFR<60 ml/min/1.73 m^2^)—MDRD equation	NA	0.83	NR	Apparent	Logistic
									0.87	NR	Split-sample	
Jardine et al. 2012 [27]—ADVANCE Major Final model	20 countries/multi-ethnic	Prospective cohort/population-based	21	Sex, eGFR, ACR, SBP, HbA1c, diabetic retinopathy, age at completion of formal education	11,140/166	Mean: 66 (SD: 6)	Doubling of serum creatinine to ≥2.26 mg/dl (≥200 µmol/l), renal replacement therapy, or renal death in diabetes	5	0.87	HL χ^2^ = 1.5 (*p* = 0.9)	Bootstrap	Cox
Jardine et al. 2012 [27]—ADVANCE Major eGFR model	20 countries/multi-ethnic	Prospective cohort/population-based	1	eGFR	11,140/166	Mean: 66 (SD: 6)	Doubling of serum creatinine to ≥2.26 mg/dl (≥200 µmol/l), renal replacement therapy, or renal death in diabetes	5	0.78	NR	Apparent	Cox
Jardine et al. 2012 [27]—ADVANCE Major ACR model	20 countries/multi-ethnic	Prospective cohort/population-based	1	ACR	11,140/166	Mean: 66 (SD: 6)	Doubling of serum creatinine to ≥2.26 mg/dl (≥200 µmol/l), renal replacement therapy, or renal death in diabetes	5	0.75	NR	Apparent	Cox
Jardine et al. 2012 [27]—ADVANCE Major eGFR+ACR model	20 countries/multi-ethnic	Prospective cohort/population-based	2	eGFR, ACR	11,140/166	Mean: 66 (SD: 6)	Doubling of serum creatinine to ≥2.26 mg/dl (≥200 µmol/l), renal replacement therapy, or renal death in diabetes	5	0.82	HL χ^2^ = 6.1 (*p* = 0.7)	Apparent	Cox
Jardine et al. 2012 [27]—ADVANCE Albuminuria Final model	20 countries/multi-ethnic	Prospective cohort/population-based	21	Ethnicity, eGFR, ACR, SBP, HTN treatment, HbA1c, diabetic retinopathy, waist circumference	7,377/2,715	Mean: 66 (SD: 6)	New-onset albuminuria in diabetes	5	0.65	HL χ^2^ = 16.5 (*p* = 0.06)	Bootstrap	Cox
Jardine et al. 2012 [27]—ADVANCE Albuminuria eGFR model	20 countries/multi-ethnic	Prospective cohort/population-based	1	eGFR	7,377/2,715	Mean: 66 (SD: 6)	New-onset albuminuria in diabetes	5	0.54	NR	Apparent	Cox
Jardine et al. 2012 [27]—ADVANCE Albuminuria ACR model	20 countries/multi-ethnic	Prospective cohort/population-based	1	ACR	7,377/2,715	Mean: 66 (SD: 6)	New-onset albuminuria in diabetes	5	0.63	NR	Apparent	Cox
Jardine et al. 2012 [27]—ADVANCE Albuminuria eGFR+ACR model	20 countries/multi-ethnic	Prospective cohort/population-based	2	eGFR, ACR	7,377/2,715	Mean: 66 (SD: 6)	New-onset albuminuria in diabetes	5	0.63	HL χ^2^ = 78.1 (*p*<0.001)	Apparent	Cox

ADVANCE, Action in Diabetes and Vascular Disease: Preterax and Diamicron MR Controlled Evaluation; ARIC, Atherosclerosis Risk in Communities Study; BMI, body mass index; CHF, congestive heart failure; CHS, Cardiovascular Health Study; CRP, c-reactive protein; DBP, diastolic blood pressure; HDL, high-density lipoprotein cholesterol; HL, Hosmer-Lemeshow; HTN, hypertension; Hx, history; KD, kidney disease; NA, not applicable; NR, not reported; NSAID, nonsteroidal anti-inflammatory drug; PVD, peripheral vascular disease; SBP, systolic blood pressure; SD, standard deviation; SLE, systemic lupus erythematosus; SNP, single nucleotide polymorphism; T2DM, type 2 diabetes mellitus;.

a Time horizon is the time over which the prediction of outcomes is made, and is the duration of follow-up in each study unless specified otherwise.

#### Populations, outcomes, and risk factors

The majority of the 30 CKD risk models were developed from samples that mostly included white individuals, and only four studies included exclusively Asian participants [23]–[26]. The number of participants included in the studies ranged from 534 to 1.6 million, and their ages ranged from 18 to 90 y. The length of follow-up in the cohort studies ranged from 1 to 10 y.

The definition of CKD was fairly consistent across prediction models (eGFR<60 ml/min/1.73 m^2^), although nine models focused on predicting diabetic nephropathy [22], and another on CKD prediction among HIV-positive individuals [26]. The included risk models used the Modification of Diet in Renal Disease (MDRD) Study equation to estimate GFR, with the exception of models from the ADVANCE study [27], which used estimates from the Chronic Kidney Disease Epidemiology Collaboration (CKD-EPI) equation. The original MDRD equation is eGFR (ml/min/1.73 m^2^) = 175×standardized Scr (mg/dl)^−1.154^×age (y)^−0.203^×1.212 [if black]×0.742 [if female], where Scr is serum creatinine [28]. The less used CKD-EPI equation is eGFR (ml/min/1.73 m^2^) = 141×min(Scr/κ, 1)^α^×max(Scr/κ, 1)^−1.209^× 0.993^age^×1.018 [if female]×1.159 [if black], where Scr is serum creatinine, κ is 0.7 for females and 0.9 for males, α is −0.329 for females and −0.411 for males, min indicates the minimum of Scr/κ or 1, and max indicates the maximum of Scr/κ or 1 [29].

Ten studies provided usable data on the numbers of candidate variables tested for inclusion in the models. This number ranged from one to 24, giving conservative estimates of the ratio of the number of observed events (outcome of interest) to the number of candidate variables ranging from six to 166. The predictors most commonly included in the final prediction models were age, sex, body mass index, diabetes status, systolic blood pressure, serum creatinine, a measure of proteinuria, and serum albumin or total protein (Table S1). Three studies used novel biomarkers or genetic or circulating factors [22],[30],[31]. Eighteen models were derived using logistic regressions, and three using Cox regressions. All studies reported the original model with beta coefficients, and five studies presented additional point-based scoring systems [21],[27],[32], or risk calculators [33],[34].

#### Performance of risk prediction models

Table 1 shows the performance of the various CKD risk models. All the included studies reported a C-statistic ranging from 0.57 to 0.88, indicating a modest-to-good discriminatory performance. Nine risk scores were internally validated, through split-sample validation in four cases (three of these were also externally validated), and bootstrapping in five other studies. Twelve risk models had an estimate of calibration: Hosmer-Lemeshow test statistics in most cases, which generally indicated good calibration.

#### CKD model improvement

Four studies assessed model improvement subsequent to adding extra variables. One study reported a significant improvement after adding circulating biomarkers (aldosterone and homocysteine) to traditional CKD risk factors [30]; the difference in AUC was 0.012 (*p* = 0.00233), NRI 6.9% (*p* = 0.0004), and IDI 0.013 (*p* = 0.004). The second study reported an AUC difference of 0.001 (*p* = 0.2) for adding genotypic information (16 single nucleotide polymorphisms) to known risk factors [31]. The third study reported no statistically significant improvement from adding uric acid, postprandial glucose, hemoglobin A_1c_, and proteinuria ≥ 100 mg/dl to traditional risk factors, with nonsignificant differences in AUC (−0.003), NRI (−0.0889), and IDI (0.0141) [25]. The last study found that a model for predicting major renal events using eGFR and albumin/creatinine ratio (ACR) (AUC: 0.818) was superior to models with either of the predictors alone (AUC: 0.779 for eGFR, and 0.752 for ACR); all three models were inferior to an expanded model with five additional variables (AUC: 0.847) (all *p*<0.05 for AUC comparison) [27]. In the same study, the eGFR+ACR (AUC: 0.629) and ACR alone (AUC: 0.627) models had similar performance for predicting new-onset albuminuria; both were superior to the eGFR alone model (AUC: 0.543) (both *p*<0.05), while all three were inferior to an extended model (AUC: 0.647) with six extra variables (all *p*<0.05 for AUC comparison) [27].

#### Validation of CKD risk prediction models

Table 2 shows the results of the external validation of CKD risk models. Only eight of the models were externally validated. Of these, only four models were validated more than once: twice for three models [25],[34]–[36] and three times for one model [21],[37],[38]. The AUC in validation studies (0.57 to 0.88) was generally lower than that in the derivation sample; the change from the original C-statistic from when the model was first derived ranged from −0.2 to +0.06 (Table 2), being negative or null except in two cases of validation of one score where it was positive [25], thus indicating a generally lower discrimination in validation populations. In the validation populations, the calibration was also poorer, though it was not assessed in most of validation studies.

**Table 2:** External validation of risk prediction models for chronic kidney disease.

Study	Name of the Model Validated	Validation Population/Country	Ethnicity	Design/Setting	Sample Size	Age (Years)	Time Horizon (Years)	Discrimination AUC	Change from the Original AUC during Development	Calibration	Reclassification	
											NRI	IDI
Bang et al. 2007 [21]	SCORED score	ARIC cohort/US	Mixed	Prospective cohort/population-based	12,038	45–65		0.71	−0.17	NR	NA	NA
Bang et al. 2008 [37]	SCORED score	NHANES 2003–2004 survey/ARIC/CHS cohort/US	Mixed	Cross-sectional (NHANES)/population-based	4,298	≥20	NA	0.88	0	NR	NA	NA
				Prospective cohort (ARIC/CHS)/population-based	21,221	≥45	5	0.78–0.80	−0.10 to −0.08			
Bang et al. 2009 [38]	SCORED score	ENRICHD and VISP cohort/US	Mixed/mainly White	Cross sectional/population-based	2,145 for ENRICHD	Mean: 61	NA	0.75	−0.13	NR	NA	NA
					3,640 for VISP	Mean: 66	NA	0.68	−0.2			
Chien et al. 2010 [25]	ARIC/CHS score 1	Chinese/Taiwan	Asian	Prospective cohort/population-based	5,168	Mean: 51.2	4	0.65	−0.03	NR	NA	NA
Chien et al. 2010 [25]	ARIC/CHS score 2	Chinese/Taiwan	Asian	Prospective cohort/population-based	5,168	Mean: 51.2	4	0.65	−0.05	NR	NA	NA
Chien et al. 2010 [25]	ARIC/CHS score 1	Chinese/Taiwan	Asian	Prospective cohort/population-based	5,168	Mean: 51.2	4	0.74	+0.06	NR	NA	NA
Chien et al. 2010 [25]	ARIC/CHS score 2	Chinese/Taiwan	Asian	Prospective cohort/population-based	5,168	Mean: 51.2	4	0.74	+0.04	NR	NA	NA
Hippisley-Cox and Coupland 2010 [34]	QKidney score	THIN Cohort/UK	Mixed—white, black, South-Asian, Chinese	Prospective cohort/population-based	1,581,745	35–74	NR	CKD: 0.88, women; 0.88, men/ESRD: 0.82, women; 0.84, men	CKD: 0, women; 0, men/ESRD: 0.02, women; 0.01, men	NR	NA	NA
Collins and Altman 2012 [36]	QKidney score	THIN Cohort/UK	Mixed—white, black, South-Asian, Chinese	Prospective cohort/population-based	1,6000,000	35–74	6.21	0.86 for CKD	−0.02	NR	NA	NA
								0.83 for ESRD	−0.01			
Chien et al. 2010 [25]	Taiwan score 2	Chin-Shan Community Cardiovascular Cohort/China	Chinese	Prospective cohort/population-based	3,205	NR	2.2	0.67	−0.1	HL test (*p* = 0.85)	−0.0889 for adding biochemical factors	0.0141 for adding biochemical factors
Blech et al. 2011 [22]	Israel score 2	Independent (Ashkenazi Jews)/Israel	White	Cross-sectional/clinic-based	906	NR	—	0.57	−0.06	NR	NA	NA
O'Seaghdha et al. 2012 [32]	Framingham score 4c	ARIC/US	White and black	Prospective cohort/population-based	1,777	Mean: 62.4	8.5	0.75 in black individuals (*n* = 424)	−0.04	HL test (*p* = 0.29)	NA	NA
								0.74 in white individuals (*n* = 1,353)	−0.05	HL test (*p* = 0.01)		
Kwon et al. 2012 [24]	Korean risk score	Korean Genome Epidemiology Study cohort/Korea	Asian	Cross-sectional/population-based	8,166	≥30	NA	0.78	−0.09	NR	NA	NA

ARIC, Atherosclerosis Risk in Communities Study; CHS, Cardiovascular Health Study; HL, Hosmer-Lemeshow; NA, not applicable; NHANES, National Health and Nutrition Examination Survey; NR, not reported; THIN, The Health Improvement Network.

### Risk Scores for Predicting Progression of CKD to ESRD

Table 3 shows the models for the prediction of progression to later stages among people with already established CKD. We found 17 CKD progression risk scores, developed from Cox regression models using data from clinical settings, mainly in white populations. Two of the CKD progression risk scores were developed from a cohort of people with type 2 diabetes and nephropathy [39],[40], and three other scores used cohorts of people exclusively with IgA nephropathy [41]–[43]. The risk factors included in CKD progression risk models varied. The number of candidate variables tested for inclusion in the models ranged from ten to 24, corresponding to a ratio of number of observed events (outcome of interest) to number of candidate variables of four to 16. For one risk model, the performance in the derivation sample was not reported [39], although the performance of the score was later assessed in a validation study conducted in a different population. When evaluated, the C-statistic of these models ranged from 0.56 to 0.94, and calibration (reported for two models only) was good. In addition to reporting beta coefficients for regression models, four studies also provided a point-based scoring system [42]–[44] or a risk calculator [45].

**Table 3:** Development of risk models for predicting the progression of chronic kidney disease.

Study	Country/Ethnicity	Design/Setting	Candidate Variables (*n*)	Risk Factors Included	*n* Total/*n* Outcomes	Age (Years)	Outcomes	Time Horizon (Years)^a^	Discrimination (AUC)	Calibration	Method of Internal Validation	Type of Model
Keane et al. 2006 [39]	28 countries (Asia, Europe, America)/mainly white	Prospective cohort/clinic-based	29	Urinary ACR, serum albumin, serum creatinine, hemoglobin	1,513 with T2DM and nephropathy/341	31–70	ESRD (need for long-term dialysis or renal transplantation)	3.4	NR	NR	None	Cox
Kent et al. 2007 [50]	Multiple countries (Asia, Europe, North America)/mainly white	Prospective cohort/clinic-based	NR	Age, gender, serum creatinine, urinary protein excretion, SBP	1,860	Mean: 51.9	kidney disease progression (doubling in serum creatinine from baseline or kidney failure, defined as onset of long-term dialysis)		0.83	HL test (*p* = 0.33)	Apparent	Cox
Wakai et al. 2006 [43]	Japan/Asian	Prospective cohort/clinic-based	NR	Age, sex, SBP, proteinuria, hematuria, serum total protein, histological grade	2,269 people with IgA nephropathy/207	5–80	ESRD (initiation of dialysis therapy)	7	0.94	NR	Apparent	Cox
									0.95/0.93	NR	Linear interpolation/Bootstrap	
Goto et al. 2009 [41]	Japan/Asian	Prospective cohort/clinic-based	17	Proteinuria, hypoalbuminemia, mild hematuria, serum total protein levels, DBP, histological grade	790 people with IgA nephropathy/68	>13	Renal deterioration including chronic dialysis	10	0.83, decision tree; 0.81, logistic	NR	Apparent	Decision tree and logistic
									0.82, decision tree	NR	Bootstrap	
Goto et al. 2009 [42]	Japan/Asian	Prospective cohort/clinic-based	10	Sex, age, SBP, proteinuria, mild hematuria, serum albumin, GFR, histological grade	2,283 people with IgA nephropathy/252	5–80	ESRD (initiation of dialysis therapy)	10	0.94	NR	Apparent	Cox
									0.94	NR	Bootstrap	
Johnson et al. 2008 [44]	US/mixed	Retrospective cohort/clinic-based	6	Age, sex, eGFR, diabetes, anemia, hypertension	9,782/323	30–89	ESRD (renal replacement therapy in patients with stage 3 or 4 CKD [MDRD defined])	5	NR	HL test (*p* = 0.99)	Apparent	Cox
									0.89	NR	Bootstrap	
Hallan et al. 2009 [47]	Norway/white	Prospective cohort/population-based	19	Age, gender, physical activity, diabetes, SBP, antihypertensive medication, HDL, eGFR, ACR	65,589/124	Mean: 50.1	ESRD (MDRD defined)	10.3	0.86	NR	Apparent	Cox
Landray et al. 2010 [45]—Landray model	England/mainly white	Prospective cohort/clinic-based	22	Creatinine level, phosphate level, urinary ACR, female sex (ERSD prediction)/NT-pro-BNP, age, current smoking, increased TnT (death prediction)	382/190 (ERSD) and 150 (death)	Mean: 61.5	ESRD (initiation of maintenance dialysis therapy or kidney transplant) or death	4.1 for ESRD and 6 for death	0.87 for ESRD and 0.82 for death	NR	Apparent	Cox
Tangri et al. 2011 [33]—Tangri model 1	Canada/mixed, mainly white	Prospective cohort/clinic-based	24	Age, sex	3,449/386	Mean: 70	Kidney failure (need for dialysis or preemptive kidney transplantation) in patients with stage 3 to 5 CKD (MDRD defined)	5	0.56	NR	Apparent	Cox
Tangri et al. 2011 [33]—Tangri model 2	Canada/mixed, mainly white	Prospective cohort/clinic-based	24	Age, sex, eGFR	3,449/386	Mean: 70	Kidney failure (need for dialysis or preemptive kidney transplantation) in patients with stage 3 to 5 CKD (MDRD defined)	5	0.89	Modified HL test (*p*<0.001)	Apparent	Cox
Tangri et al. 2011 [33]—Tangri model 3	Canada/mixed, mainly white	Prospective cohort/clinic-based	24	Age, sex, eGFR, albuminuria	3,449/386	Mean: 70	Kidney failure (need for dialysis or preemptive kidney transplantation) in patients with stage 3 to 5 CKD (MDRD defined)	5	0.91	Modified HL test (*p*<0.001)	Apparent	Cox
Tangri et al. 2011 [33]—Tangri model 4	Canada/mixed, mainly white	Prospective cohort/clinic-based	24	Age, sex, eGFR, albuminuria, diabetes, hypertension	3,449/386	Mean: 70	Kidney failure (need for dialysis or preemptive kidney transplantation) in patients with stage 3 to 5 CKD (MDRD defined)	5	0.91	NR	Apparent	Cox
Tangri et al. 2011 [33]—Tandri model 5	Canada/mixed, mainly white	Prospective cohort/clinic-based	24	Age, sex, eGFR, albuminuria, SBP, DBP, weight	3,449/386	Mean: 70	Kidney failure (need for dialysis or preemptive kidney transplantation) in patients with stage 3 to 5 CKD (MDRD defined)	5	0.92	NR	Apparent	Cox
Tangri et al. 2011 [33]—Tangri model 6	Canada/mixed, mainly white	Prospective cohort/clinic-based	24	Age, sex, eGFR, albuminuria, serum albumin, serum phosphate, serum bicarbonate, serum calcium	3,449/386	Mean: 70	Kidney failure (need for dialysis or preemptive kidney transplantation) in patients with stage 3 to 5 CKD (MDRD defined)	5	0.92	Modified HL test (*p*<0.001)	Apparent	Cox
Tangri et al. 2011 [33]—Tangri model 7	Canada/mixed, mainly white	Prospective cohort/clinic-based	24	Age, sex, eGFR, albuminuria, serum albumin, diabetes, hypertension, SBP, DBP, serum phosphate, serum bicarbonate, serum calcium	3,449/386	Mean: 70	ESRD or death	5	0.92	NR	Apparent	Cox
Desai et al. 2011 [40]—TREAT ESRD model 1	US and Canada/mainly white, but also black and other	Prospective cohort (people with T2DM, CKD, and anemia)/clinic-based	35	Age, sex, race, BMI, insulin use, eGFR, serum urea nitrogen, urinary protein-creatinine ratio, hemoglobin level, Hx of heart failure, Hx of stroke, Hx of PAD, Hx of arrhythmia, serum ferritin, CRP, Hx of AKI	995/222 ESRD (407 death or ESRD)	Mean: 68	ESRD or death	3.5	0.84 for ESRD, and 0.75 for ESRD or death	NR	Apparent	Cox
Desai et al. 2011 [40]—TREAT ESRD model 2	US and Canada/mainly white, but also black and other	Prospective cohort (people with T2DM, CKD and anemia)/clinic-based	37	Age, sex, race, BMI, insulin use, eGFR, serum urea nitrogen, urinary protein-creatinine ratio, hemoglobin level, Hx of heart failure, Hx of stroke, Hx of PAD, Hx of arrhythmia, serum ferritin, CRP, Hx of AKI, TnT, NT-pro-BNP	995/222 ESRD (407 death or ESRD)	Mean: 68	ESRD or death	3.5	0.85 for ESRD, and 0.76 for ESRD or death	NR	Apparent	Cox

AKI, acute kidney injury; BMI, body mass index; DBP, diastolic blood pressure; HDL, high-density lipoprotein cholesterol; HL, Hosmer-Lemeshow; Hx, history; NT-pro-BNP, N-terminal pro–brain natriuretic peptide; PAD, peripheral arterial disease; NR, not reported; SBP, systolic blood pressure; T2DM, type 2 diabetes mellitus; TnT, Troponin T.

a Time horizon is the time over which the prediction of outcomes is made, and is the duration of follow-up in each study unless specified otherwise.

As shown in Table 4, five of the CKD progression risk models were externally validated (C-statistic: 0.83 to 0.91); the change in C-statistic from the original value when the model was first developed ranged from −0.1 to +0.03. This change was negative in all but one case, thus indicating a generally poorer discrimination.

**Table 4:** External validation of risk models for predicting chronic kidney disease progression.

Study	Name of the Model Validated	Country/Ethnicity	Design/Setting	Sample Size	Mean Age (Years)	Time-Horizon (Years)	Discrimination AUC	Change from Original AUC	Calibration	Reclassification	
										NRI	IDI
Landray et al. 2010 [45]	Landray model	England/mainly white	Prospective cohort/clinic-based	213	61.5	4.1 for ESRD, 6 for death	0.91 for ESRD, 0.82 for death	+0.03 for ESRD, 0 for death	NR	NA	NA
Tangri et al. 2011 [33]	Tangri model 2	Canada/mixed, mainly white	Prospective cohort/clinic-based	4,942	69	5	0.79	−0.1	NR	NR	NR
Tangri et al. 2011 [33]	Tangri model 3	Canada/mixed, mainly white	Prospective cohort/clinic-based	4,942	69	5	0.83	−0.08	NR	NR	0.10 (42%) model 3 versus model 2
Tangri et al. 2011 [33]	Tangri model 6	Canada/mixed, mainly white	Prospective cohort/clinic-based	4,942	69	5	0.84	−0.08	Modified HL (Nam and D'Agostino) statistic: 32	NR	0.02 (6%) model 3 versus model 6
Tangri et al. 2011 [33]	Tangri model 7	Canada/mixed, mainly white	Prospective cohort/clinic-based	4,942	69	5	0.84	−0.08	NR	NR	NR
Desai et al. 2011 [40]	REENAL risk score	US and Canada/mainly white	Prospective cohort/clinic-based	995	68	3.5	0.80 (0.81 when Troponin T and N-terminal pro–brain natriuretic peptide added to original model)	NA (as original AUC not reported)	NR	19 when Troponin T, and N-terminal pro–brain natriuretic peptide added to original model	NR

HL, Hosmer-Lemeshow; NA, not applicable; NR, not reported.

Two studies investigated the improvement of three different CKD progression models [33],[40], after adding biomarkers to traditional risk factors (serum bicarbonate and phosphate in one case [33], and Troponin T plus brain natriuretic peptide in the two other cases) [40]. The change in C-statistic or AUC varied from 0.01 to 0.02, and NRI from 16.9% to 26.7%.

### Impact Studies and Incorporation of CKD Prediction Models in Clinical Practice Guidelines

We found no evidence in guidelines of recommendations for using CKD risk prediction models to estimate the risk in patients either in clinical or community settings. We also did not find any studies assessing the impact of adopting CKD (occurrence and progression) risk scores in clinical practice on the process of care and outcomes of patients.

## Discussion

This systematic review shows that a sizeable number of renal risk prediction models have been developed, with, however, variation in their quality. Reasons for this may be specific to nephrology, where risk prediction is still in its infancy and the methodology for predictive research may be underappreciated. Despite the heterogeneity of CKD, with several specific forms, this review demonstrates the feasibility of defining individual renal risk using a combination of commonly assessed variables. Indeed, there was remarkable similarity between the variables that entered the prediction models (Tables S1 and S2), each developed in a distinct group of participants, sometimes with specific forms of CKD. The discriminative performance of existing models was generally acceptable-to-good on the derivation sample. However, when corrected for overfitting (internal validation) or tested in a new population (external validation), this discriminative performance was modest-to-acceptable. For CKD risk prediction, the SCORED model appears to be the most reliable, as it is the most externally validated model, with a reasonable discrimination [21]. Regarding CKD progression, no risk model has been extensively validated in different populations.

### Potential Public Health and Clinical Applications of CKD Risk Models

Risk prediction models have potential applications in the prevention and management of CKD. Risk communication to patients may motivate them for lifestyle modification and adherence to prescribed therapies. Using models for predicting progression of CKD, clinicians may be able to tailor disease-modifying therapies as well as frequency of monitoring to individual risk. Indeed, therapies for controlling several variables included in CKD progression models (e.g., diabetes and hypertension) have been shown to delay CKD progression. Furthermore, using CKD progression models to identify patients who are most likely to need renal replacement therapy would allow patient education on available therapeutic options. CKD risk scores may be useful in the assessment of novel technologies or biomarkers for risk prediction, or for patient recruitment in prevention trials. They can also serve in mass screening and public education initiatives. For all these applications, estimates of CKD risk from prediction models must be accurate and validated.

### Development of Existing CKD Risk Prediction Models

The performance of prediction models is largely determined by the appropriateness of the methodological approaches used to develop them. Virtually none of the existing CKD models was developed using data specifically collected for risk modeling purposes. This may raise concerns about the quality of the predictors and outcomes tested/included in the models, as well as the completeness of measurements. Lessons learned from CVD prediction suggest that the source of data for model development matters less, provided that the ensuing model can reliably predict the outcome of interest in different populations [46]. Indeed, in practice, assembling data only for the purpose of modeling can be challenging, and researchers tend to rely on available data collected for other reasons [9]. At least four of the models were likely statistically underpowered, based on having a ratio of the number of outcomes to the number of candidate predictors of <8 [24],[26],[40],[41],[47]. The performance of such models tends to drop substantially when the model is applied to different populations [24]. Other mistakes that affect model performance were present across studies, including dichotomization of continuous variables prior to modeling, linearity assumptions without formal testing, and exclusion of participants with missing values on predictor/outcome variables.

### Internal Validation of Existing CKD Prediction Models

One model was published without indicators of performance during the derivation process [39]. Most models provided measures of performance, which were based on the direct application of the model to the derivation sample (apparent performance). This approach is optimistic (self-fulfilling prophecy). Some models provided performance measures from internal split-sample or bootstrap validation, which may provide the new user with an idea about what to expect when applying the model to different populations. When reported, discrimination was always good for CKD progression models, and acceptable-to-good for prevalent/incident CKD models, indicating that these models were able to differentiate participants with CKD from those without in the derivation sample. Calibration, a key property of model performance, was less commonly assessed during the derivation process. Whether calibration performance of a model in one population can inform its behavior in another population is still debated. However, there is a growing agreement that, because calibration is largely affected by the background risk, which varies across populations, models need to be updated through recalibration procedures to provide accurate estimates of the risk in new populations. There have been attempts to update some of the existing CKD models, but the procedures used (addition of extra variables) have focused on improvement in discriminatory performance [25],[27],[30],[31], and only one study reported change in the calibration properties [27].

### External Validation of Existing CKD Risk Prediction Models

The demonstration of the performance of a model in new populations is an important step before recommending its widespread use. A limited number of existing CKD prediction models have been tested on different populations [21],[22],[25],[32]–[35],[45]. Validation studies have mainly been conducted by the same group of investigators who developed the models. This is methodologically inferior and quantitatively insufficient to provide good indicators of models' behavior in various populations. Hence, more validation studies of existing models are needed, ideally by different investigators, to guarantee their generalizability to a larger number of people. Instead of developing new models for their own setting, investigators in the field of CKD may consider integrating aspects of the validation of existing models into future studies. In addition to providing indicators of the performance of existing models in various settings, such an approach limits unnecessary development of new models.

### Implementation of Existing CKD Prediction Models

CKD models have largely been published in the form of mathematical equations, with point-scoring systems [21],[32],[42]–[44] or calculators [33],[34],[45] for a few. The mathematical format may not be suitable for application in various settings, particularly by busy clinicians who may be less familiar with manipulating complex formulas. Translation efforts are therefore needed to convert accurate and validated CKD prediction equations into simple tools that can improve their uptake in various settings [33]. Some context-specific efforts may also be required to derive appropriate cutoffs for defining high-risk status when models are integrated in guidelines for screening. It is, however, important to confirm whether the implementation of CKD risk prediction models affects the behavior of healthcare providers and improves outcomes of care. At present, no implementation study of CKD risk prediction models has been conducted.

Published studies have relied on GFR estimated from the MDRD equation to define CKD [28]. The MDRD equation provides less accurate estimates of GFR in different ethnic groups, compared with estimates derived from the more recent CKD-EPI equation [29], resulting in “over-diagnosis” of CKD using the MDRD equation. There have been suggestions that this over-diagnosis may have little effect on estimates of the association between risk factors and CKD outcomes [24],[32] and, accordingly, on discriminatory performance when models developed to predict the outcome of CKD based on the MDRD equation are applied to the outcome of CKD based on the CKD-EPI formula. However, the difference in prevalence/incidence of CKD based on the two formulas will invite recalibration of MDRD equation–based models to improve their applicability with the increasing international adoption of CKD-EPI estimates of GFR for CKD diagnosis.

Participants in the reviewed studies were overwhelmingly white. A homogenous population does not allow researchers to probe into the whole scope of the variability in CKD risk. This is even more important for CKD than for other diseases, as some ethnic groups are particularly prone to CKD (e.g., African-Americans), and the use of risk stratification tools in these groups may be more warranted. Future studies should therefore incorporate more participants of different ethnic backgrounds.

### Strengths and Limitations of the Review

The strengths of this review include the exclusion of studies that reported only effect estimates for independent association of risk factors with CKD. These measures alone provide no information on model calibration and global discriminative performance. The case for predictive testing depends not merely on the magnitude of the risk ratio, but also on the extent to which the test results are useful for improving prediction of disease when various risk factors are accounted for. This systematic review may also help policy makers decide whether to incorporate risk tools in guidelines for screening, routine evaluation, and management of CKD. Such an inclusion may be premature at this point in time, particularly in the absence of extensive external validation studies and impact analyses. We did not explicitly rank or categorize the quality of existing CKD risk models, mindful that there is no agreed-on scientific system for rating risk prediction model quality. Some will argue that minimizing risk for potential bias is of critical importance, while others might support the view that a risk score should be judged on its ability to perform accurately across diverse settings. Finally, our ability to assess publication bias was limited.

### Conclusion

This review suggests that risk models for predicting CKD or its progression have a modest-to-acceptable discriminatory performance, but would need to be better calibrated and externally validated—and the impact of their use on outcomes assessed—before these are incorporated in guidelines. Their potential application for screening or management to identify CKD in a heterogeneous population will also depend on the context. In the US, for example, the adoption of the Kidney Disease Outcomes Quality Initiative guidelines has led to systematic reporting of eGFR by laboratories whenever serum creatinine is requested. Consequently, a certain degree of de facto opportunistic CKD screening is happening. In such a context, risk scores for predicting CKD progression or outcomes would be particularly useful for defining prognosis in identified people. However, an important fraction of the population at high risk of CKD without access to care could still be identified in the community using CKD risk prediction tools.

## Supporting Information

Table S1 **Factors included in models of risk prediction for chronic kidney disease.**
(DOC)

Table S2 **Factors included in risk models for predicting the progression of chronic kidney disease.**
(DOC)

Text S1 **PRISMA checklist.**
(DOC)

Text S2 **Search terms for risk model development or validation studies.**
(DOC)

Text S3 **Search terms for impact studies.**
(DOC)

## Abbreviations

ACR: albumin/creatinine ratio
ADVANCE: Action in Diabetes and Vascular Disease: Preterax and Diamicron MR Controlled Evaluation
AUC: area under the receiver operating characteristic curve
CKD: chronic kidney disease
CKD-EPI: Chronic Kidney Disease Epidemiology Collaboration
CVD: cardiovascular disease
eGFR: estimated glomerular filtration rate
ESRD: end-stage renal disease
GFR: glomerular filtration rate
IDI: integrated discrimination improvement index
MDRD: Modification of Diet in Renal Disease
NRI: net reclassification improvement
