# Supplementary material for: Risk Models to Predict Chronic Kidney Disease and Its Progression: A Systematic Review
Source: PLoS Med. 2012 Nov 20;9(11):e1001344. doi: 10.1371/journal.pmed.1001344 (PMC3502517; doi:10.1371/journal.pmed.1001344)
Supplement: Table S1 — Factors included in models of risk prediction for chronic kidney disease. (DOC) [file pmed.1001344.s001.doc]

Table S4: Factors included in models of risk prediction for chronic kidney disease

| **Author, Reference** | **Name of the risk model** | **Age** | **Sex/**  **gender** | **Ethnicity** | **Anemia/**  **Hemoglobin level** | **Estimated glomerular filtration rate** | **Proteinuria**  **/albuminuria** | **Glucose level/**  **Diabetes/**  **history of diabetes** | **Blood pressure/ Hypertension** | **Cardiovascular disease** | **Smoking** | **Blood**  **lipids s** | **Body mass**  **index/**  **adiposity** | **Kidney stone / Uric acid** | Aldosterone | **Homocysteine** | **Genes** | **Diabetic retinopathy** |
| --- | --- | --- | --- | --- | --- | --- | --- | --- | --- | --- | --- | --- | --- | --- | --- | --- | --- | --- |
| Bang et al, 2007 | SCORED score | Yes | Yes | No | Yes | No | No | Yes | Yes | Yes | No | No | No | No | No | No | No | No |
| Kshirsagar et al, 2008 | ARIC/CHS score 1 | Yes | Yes | No | Yes | No | No | Yes | Yes | Yes | No | No | No | No | No | No | No | No |
| Kshirsagar et al, 2008 | ARIC/CHS score 2 | Yes | Yes | No | Yes | No | No | Yes | Yes | Yes | No | Yes | No | No | No | No | No | No |
| Fox et al, 2010 | Framingham score 1 | Yes | Yes | No | No | No | No | No | No | No | No | No | No | No | No | No | No | No |
| Fox et al, 2010 | Framingham score 2 | Yes | Yes | No | No | Yes | No | Yes | Yes | No | Yes | Yes | No | No | No | No | No | No |
| Fox et al, 2010 | Framingham score 3 | Yes | Yes | No | No | Yes | No | Yes | Yes | No | Yes | Yes | Yes | No | Yes | Yes | No | No |
| Hippisley-Cox et al, 2010 | QKidney score | Yes | Yes | Yes | No | No | No | Yes | Yes | Yes | Yes | Yes | Yes | Yes | No | No | No | No |
| Chien, et al 2010 | Taiwan score 1 | Yes | Yes | No | No | No | No | Yes | Yes | Yes | No | No | Yes | No | No | No | No | No |
| Chien, et al 2010 | Taiwan score 2 | Yes | Yes | No | No | No | Yes | Yes | Yes | Yes | No | No | Yes | Yes | No | No | No | No |
| Halbesma et al, 2011 | PREVEND score | Yes | Yes | No | No | Yes | Yes | No | Yes | No | No | No | No | No | No | No | No | No |
| Ando et al, 2011 | Japan/HIV score | Yes | Yes | No | No | Yes | Yes | Yes | No | No | No | No | No | No | No | No | No | No |
| Blech et al, 2011 | Israel score 1 | Yes | Yes | No | No | No | No | Yes | No | No | No | No | No | No | No | No | No | No |
| Blech et al, 2011 | Israel score | No | Yes | Yes | No | No | No | Yes | No | No | No | No | No | No | No | No | Yes | No |
| Thakkinstian etal, 2011 | Thailand score | Yes | No | No | No | No | No | Yes | Yes | No. | No | No | No | Yes | No | No | No | No |
| O’Seaghdha et al, 2012 | Framingham score 3a | Yes | Yes | No | No | Yes | Yes | Yes | Yes | No | No | No | No | No | No | No | Yes | No |
| O’Seaghdha et al, 2012 | Framingham score 3b | Yes | Yes | No | No | No | No | No | No | No | No | No | No | No | No | No | Yes | No |
| O’Seaghdha et al, 2012 | Framingham score 4a | Yes | No | No | No | No | No | Yes | Yes | No. | No | No | No | No | No | No | No |  |
| O’Seaghdha et al, 2012 | Framingham score 4b | Yes | No | No | No | Yes | No | Yes | Yes | No | No | No | No | No | No | No | No | No |
| O’Seaghdha et al, 2012 | Framingham score 4c | Yes | No | No | No | No | No | Yes | Yes | No | No | No | No | No | No | No | No | No |
| Alssema, et al, 2012 | Rotterdam- Hoorn score | Yes | No | No | No | No | No | Yes | Yes | Yes | No | No | Yes | No | No | No | No | No |
| Kwon et al, 2012 | Korean risk score | Yes | Yes | No | No | No | No | Yes | Yes | Yes | No | No | No | No | No | No | No | No |
| Jardine et al, 2012 | ADVANCE Major final model | Yes | Yes | No | No | Yes | Yes | Yes | Yes | No | No | No | Yes | No | No | No | No | Yes |
| Jardine et al, 2012 | ADVANCE Major eGFR model | No | No | No | No | Yes | No | No | No | No | No | No | No | No | No | No | No | No |
| Jardine et al, 2012 | ADVANCE Major ACR model | No | No | No | No | No | Yes | No | No | No | No | No | No | No | No | No | No | No |
| Jardine et al, 2012 | ADVANCE Major eGFR+ACR model | No | No | No | No | Yes | Yes | No | No | No | No | No | No | No | No | No | No | No |
| Jardine et al, 2012 | ADVANCE Albuminuria final model | No | No | Yes | No | Yes | Yes | Yes | Yes | No | No | No | No | No | No | No | No | Yes |
| Jardine et al, 2012 | ADVANCE Albuminuria eGFR model | No | No | No | No | Yes | No | Yes | No | No | No | No | No | No | No | No | No | No |
| Jardine et al, 2012 | ADVANCE Albuminuria ACR model | No | No | No | No | No | Yes | Yes | No | No | No | No | No | No | No | No | No | No |
| Jardine et al, 2012 | ADVANCE Albuminuria eGFR+ACR model | No | No | No | No | Yes | Yes | Yes | No | No | No | No | No | No | No | No | No | No |

**References**
