# Supplementary material for: Risk Models to Predict Chronic Kidney Disease and Its Progression: A Systematic Review
Source: PLoS Med. 2012 Nov 20;9(11):e1001344. doi: 10.1371/journal.pmed.1001344 (PMC3502517; doi:10.1371/journal.pmed.1001344)
Supplement: Table S2 — Factors included in risk models for predicting the progression of chronic kidney disease. (DOC) [file pmed.1001344.s002.doc]

TableS5: Risk factors included in risk models for predicting the progression of chronic kidney disease

| **Author, Reference** | **Score name/country** | **Age** | **Gender** | **Serum creatinine** | **Estimate Glomerular filtration rate** | **Urinary albumin creatinine**  **ratio** | **Serum albumin / Serum total protein** | **Urinary protein excretion** | **Blood pressure** | **Hematuria** | **Anemia/ hemoglobin level** | **Histological grade** | **Diabetes** | **Cholesterol level** | **Weight/ Body mass index** | **Physical activity** | **Serum phosphate levels** | **Serum bicarbonate** |
| --- | --- | --- | --- | --- | --- | --- | --- | --- | --- | --- | --- | --- | --- | --- | --- | --- | --- | --- |
| Keane et al, 2006 [1] | REENAL | No | No | Yes | No | Yes | Yes | No | NR | No | Yes | No | No | No | No | No | No | No |
| Kent at al, 2007 [2] | AIPRD | Yes | Yes | Yes | No | No | No | Yes | Yes | No | No | No | No | No | NO | No | No | No |
| Wakai et al, 2006 [3] | Japan | Yes | Yes | No | No | No | Yes | Yes | Yes | Yes | No | Yes | No | No | No | No | No | No |
| Goto et al, 2009 [4] | Japan | No | No | No | No | No | Yes | Yes | Yes | Yes | No | Yes | No | No | No | No | No | No |
| Goto et al, 2009 [5] | Japan | Yes | Yes | No | Yes | No | Yes | No | Yes | Yes | No | Yes | No | N | No | No | No | No |
| Johnson, et al, 2008 [6] | Canada | Yes | Yes | No | Yes | No | No | No | Yes | No | Yes | No | Yes | No | No | No | No | No |
| Hallan et al, 2009 [7] | HUNT-2 | Yes | Yes | No | Yes | Yes | No | No | Yes | No | No | No | Yes | Yes | No | Yes | No |  |
| Landray et al, 2010 [8] – | Landray Model | Yes | Yes | Yes | No | Yes | Yes | No | No | No | No | No | No | No | No | No | Yes | No |
| Tangri et al, 2010 [9] – | Tangri Model 1 | Yes | Yes | No | No | No | No | No | No | No | No | No | No | No | No | No | No | No |
| Tangri et al, 2010 [9] – | Tangri Model 3 | Yes | Yes | No | Yes | No | No | No | No | No | No | No | No | No | No | No | No | No |
| Tangri et al, 2010 [9]– | Tangri Model 3 | Yes | Yes | No | Yes | No | No | Yes | No | No | No | No | No | No | No | No | No | No |
| Tangri et al, 2010 [9]– | Tangri Model 4 | Yes | Yes | No | Yes | No | No | Yes | Yes | No | No | No | Yes | No | No | No | No | No |
| Tangri et al, 2010 [9] – | Tandri Model 5 | Yes | Yes | No | Yes | No | No | No | Yes | No | No | No | Yes | No` | Yes | No | No | No |
| Tangri et al, 2010 [9] | Tangri Model 6 | Yes | Yes | No | Yes | No | No | Yes | No | No | No | Np | No | No | Yes | No | Yes | Yes |
| Tangri et al, 2010 [9] | Tangri Model 7 | Yes | Yes | No | Yes | No | No | Yes | Yes | No | No | No | Yes | NO | Yes | No | Yes | Yes |
| Desai et al , 2011[10] | TREAT ESRD modela | Yes | Yes | Yes | Yes | Yes | No | No | No | No | Yes | No | Yes | No | Yes | No | No | No |

HUNT 2, second Nord-Trøndelag Health Study (HUNT

**References**

1. Keane WF, Zhang Z, Lyle PA, Cooper ME, de Zeeuw D, et al. (2006) Risk scores for predicting outcomes in patients with type 2 diabetes and nephropathy: the RENAAL study. Clin J Am Soc Nephrol 1: 761-767.

2. Kent DM, Jafar TH, Hayward RA, Tighiouart H, Landa M, et al. (2007) Progression risk, urinary protein excretion, and treatment effects of angiotensin-converting enzyme inhibitors in nondiabetic kidney disease. J Am Soc Nephrol 18: 1959-1965.

3. Wakai K, Kawamura T, Endoh M, Kojima M, Tomino Y, et al. (2006) A scoring system to predict renal outcome in IgA nephropathy: from a nationwide prospective study. Nephrol Dial Transplant 21: 2800-2808.

4. Goto M, Kawamura T, Wakai K, Ando M, Endoh M, et al. (2009) Risk stratification for progression of IgA nephropathy using a decision tree induction algorithm. Nephrol Dial Transplant 24: 1242-1247.

5. Goto M, Wakai K, Kawamura T, Ando M, Endoh M, et al. (2009) A scoring system to predict renal outcome in IgA nephropathy: a nationwide 10-year prospective cohort study. Nephrol Dial Transplant 24: 3068-3074.

6. Johnson ES, Thorp ML, Platt RW, Smith DH (2008) Predicting the risk of dialysis and transplant among patients with CKD: a retrospective cohort study. Am J Kidney Dis 52: 653-660.

7. Hallan SI, Ritz E, Lydersen S, Romundstad S, Kvenild K, et al. (2009) Combining GFR and albuminuria to classify CKD improves prediction of ESRD. J Am Soc Nephrol 20: 1069-1077.

8. Landray MJ, Emberson JR, Blackwell L, Dasgupta T, Zakeri R, et al. (2010) Prediction of ESRD and death among people with CKD: the Chronic Renal Impairment in Birmingham (CRIB) prospective cohort study. Am J Kidney Dis 56: 1082-1094.

9. Tangri N, Stevens LA, Griffith J, Tighiouart H, Djurdjev O, et al. (2011) A predictive model for progression of chronic kidney disease to kidney failure. JAMA 305: 1553-1559.

10. Desai AS, Toto R, Jarolim P, Uno H, Eckardt KU, et al. (2011) Association between cardiac biomarkers and the development of ESRD in patients with type 2 diabetes mellitus, anemia, and CKD. Am J Kidney Dis 58: 717-728.
