# Supplementary material for: Risk Models to Predict Chronic Kidney Disease and Its Progression: A Systematic Review
Source: PLoS Med. 2012 Nov 20;9(11):e1001344. doi: 10.1371/journal.pmed.1001344 (PMC3502517; doi:10.1371/journal.pmed.1001344)
Supplement: Text S2 — Search terms for risk model development or validation studies. (DOC) [file pmed.1001344.s004.doc]

**Text S2: Search terms for risk model development or validation studies**

**Database: PubMed (January 1980 until June 2012)**

#1 "chronic renal insufficiency" OR ("Kidney Failure, Chronic"[Mesh]) OR "chronic kidney disease" OR "chronic kidney failure"

#2 (Predict* AND Risk*) OR "decision model" OR "prognostic model"

#3 "roc curve" OR "receiver operating characteristics"

#4 "predicting" OR "predictive value of tests" OR "prediction model" OR "prediction tool" OR "prediction rule" OR "predictive model"

#5 "risk assessment" OR "risk score" OR "risk engine" OR "risk equation" OR "risk algorithm" OR "risk table" OR "risk function"

OR "risk calculator" OR "risk calculation"

#6 "validation" OR "discrimination" OR "calibration"

#7 #2 OR #3 OR #4 OR #5 OR #6

#8 #1 AND #7

**Database: EMBASE (January 1980 until June 2012)**

#1 chronic AND ('kidney'/exp OR kidney) AND failure

#2 chronic AND ('kidney'/exp OR kidney) AND ('disease'/exp OR disease)

#3 # 1 OR #2

#4 'decision model'

#5 predict* AND risk*

#6 'prognostic model'

#7 'roc curve'/exp OR 'roc curve' OR 'receiver operating characteristics'/exp OR 'receiver operating characteristics'

#8 'predicting' OR 'predictive value of tests'/exp OR 'predictive value of tests' OR 'prediction model' OR 'prediction tool' OR 'prediction rule' OR 'predictive model'

#9 'risk assessment'/exp OR 'risk assessment' OR 'risk score' OR 'risk engine' OR 'risk equation' OR 'risk algorithm' OR 'risk table' OR 'risk function' OR 'risk calculator' OR 'risk calculation'

#10 'validation' OR 'discrimination' OR 'calibration'/exp OR 'calibration'

#11 #4 OR #5 OR #6 OR #7 OR #8 OR #9 OR #10

#8 #3 AND #11 AND [humans]/lim AND [1-1-1980]/sd NOT [6-20-2012]/sd
