# Supplementary material for: Risk Models to Predict Chronic Kidney Disease and Its Progression: A Systematic Review
Source: PLoS Med. 2012 Nov 20;9(11):e1001344. doi: 10.1371/journal.pmed.1001344 (PMC3502517; doi:10.1371/journal.pmed.1001344)
Supplement: Text S3 — Search terms for impact studies. (DOC) [file pmed.1001344.s005.doc]

**Text S3: Search terms for impact studies**

Search terms used to identify all impact studies, which are combined with each specific risk scores acronym, or if not applicable the name of the cohort in which the score was developed or first author:

PubMed: (Effectiveness [tiab] OR Comparing [tiab] OR Compared [tiab] OR Evaluate [tiab]) AND (Algorithm [tiab] OR Strategy [tiab] OR Managed [tiab] OR Management [tiab] OR Decision [tiab]) AND ("chronic renal insufficiency" OR ("Kidney Failure, Chronic"[Mesh]) OR "chronic kidney disease" OR "chronic kidney failure") NOT (Animals [MeSH] NOT Humans[MeSH]).

EMBASE: (Effectiveness:ti,ab OR Comparing:ti,ab OR Compared:ti,ab OR Evaluate:ti,ab) AND (Algorithm:ti,ab OR Strategy:ti,ab OR Managed:ti,ab OR Management:ti,ab OR Decision:ti,ab) AND ("chronic renal insufficiency" OR ("Chronic kidney failure"]) OR "chronic kidney disease" OR "chronic kidney failure") AND [humans]/lim AND [1-1-1980]/sd NOT [6-20-2012]/sd
